# Understanding Risk-taking Behavior in Bullies, Victims, and Bully Victims Using Cognitive- and Emotion-Focused Approaches

**DOI:** 10.3389/fpsyg.2016.01838

**Published:** 2016-11-29

**Authors:** Kean Poon

**Affiliations:** Department of Special Education and Counselling, The Education University of Hong KongHong Kong, Hong Kong

**Keywords:** risk assessment, bullying, adolescents, peer victimization, risk taking

## Abstract

Bullying and risky behavior are two common problems among adolescents and can strongly affect a youth’s overall functioning when both coexist. Some studies suggest that bullying in adolescence may promote risky behavior as a coping strategy to deal with victimization related stress. Other studies consider bullying as an outcome of high-risk behavior. Despite the association between the two is well-established, no study has examined the risk-taking patterns among bullying groups (i.e., bully, victim, and bully victim). This study attempted to elucidate the potential relationships between bullying and risk-taking by addressing the two models: a cognitive-focused model and an emotion-focused model of risk taking, and to clarify how adolescents’ characteristics in risk taking associate with bullying outcomes.

**Method:** 136 Chinese adolescents (Mean Age = 14.5, *M* = 65, *F* = 71) were recruited and grouped according to bullying identity: Bully (*n* = 27), Victim (*n* = 20), Bully victim (*n* = 37) and Control (*n* = 52). Cognitive Appraisal of Risky Events (CARE) questionnaire was used to measure participants’ expectancies about the risks, benefits and involvement associated with risky activities. Cambridge Gambling Task (CGT) was administered to capture the emotion-laden process in risk taking.

**Results:** Cognitively, Bully was associated with an overestimation of risk while Victim was associated with an underestimation of risk and overrated benefit. Bully victim exhibited a unique pattern with an overestimation of benefit and risk. All study groups projected higher involvement in risky behavior. Behaviorally, both Bully and Bully victim were associated with high risk modulation whereas Victim was associated with impulsive decision-making. Interestingly, compared with bully, bully victim had significantly higher bullying scores, suggesting a wider range and more frequent bullying activities. In conclusion, Bully maybe a group of adolescents that is vigilant in situational deliberation and risk modulation while Victims with high impulsivity, are more likely to place themselves in risky situations. Bully victims presented the combined pattern of the two pure groups and associated with the highest risk-taking propensity. Better picture of risk taking pattern associated with different groups was illustrated, allowing better matching for future prevention and intervention program for distinct bullying individuals.

## Introduction

Bullying and victimization among youth is a serious and complex problem that is receiving increased attention. Bullying has been conceptualized as a distinct type of aggression characterized by a repeated and systematic abuse of power (Ttofi et al., 2012). It encompasses a spectrum of both physical and verbal aggressive actions. It can be direct (e.g., hitting, kicking, threatening, and extortion) or indirect (e.g., spreading rumors and social exclusion) (Karatzias et al., 2002). Because bullying involves a bully and a victim, early research tended to dichotomize children into one of these two mutually exclusive groups. However, there also appears to be a third group of bully victims who both bully and are bullied by others (Haynie, 2001; Veenstra et al., 2005).

Bullying and high-risk activities are two common problems among adolescents worldwide and can strongly affect a youth’s physical, psychological, social, and educational functioning when both coexist (Currie et al., 2012). Recently, the link between bullying and risk-taking behavior is an emergent area of research. To date, studies have found associations between bullying and delinquency (Olweus, 1979), alcohol/substance use (Kaltiala-Heino et al., 2000; Nansel et al., 2001; Carlyle and Steinman, 2007), smoking (Ellickson et al., 1997; Forero et al., 1999), and unprotected sex (Liang et al., 2007). In particular, bullies and bully victims often exhibit the highest rate of wide ranged risk-taking behaviors (Haynie, 2001). On the other hand, there are major inconsistencies in the literature examining the association between victims and risk-taking behaviors. For example, some studies have suggested that adolescent victims are at a higher risk of heavy drinking and substance abuse than non-victim adolescents are (Wills and Filer, 1996; Khantzian, 1997; Maniglio, 2009). Other studies have revealed that victims of bullying had lower levels of smoking (Forero et al., 1999) and alcohol consumption (Nansel et al., 2001; Carlyle and Steinman, 2007) when compared to controls. Studies that explain the association between bullying and risk-taking behavior are bi-directional (e.g., Collier et al., 2013). Some studies suggest that bullying or other forms of peer victimization in adolescence may promote risky behavior such as substance abuse as a coping strategy or self-medication attempting to deal with or anesthetize victimization related stress or negative feelings (Danielson et al., 2010; Durand et al., 2013; Hong et al., 2014). However, other studies consider bullying as an outcome of, rather than a risk factor for, high-risk behavior. Despite the association between bullying and risk-taking behaviors, no study to date has examined risk-taking patterns among the three bully groups.

### Risk Taking: The Decision-Making Model and the Social-Neuroscience Model

Both human and animal studies of risk-taking behavior have proposed models that predict risky choice. Recent studies have provided two new perspectives: the decision-making model (e.g., Millstein and Halpern-Felsher, 2001) and the social-neuroscience model (e.g., Steinberg, 2008). Decision-making theorists posit that individuals’ beliefs about the consequences of their actions and perceptions of their vulnerability regarding those consequences play a key role in their behavior (Millstein and Halpern-Felsher, 2001). Researchers are interested in understanding why adolescents make the decisions they do and their competence in making these decisions. In conceptualizing and measuring perceptions of risk, decision-making theorists observe whether people recognize the benefits and risks inherent in a given situation in order to measure their sense of risk and vulnerability (Slovic, 1987; Siegrist et al., 2000). Recent studies have suggested that engagement in risky activities is determined by individual differences in cognitive appraisal (expected benefit minus expected risk) (Fromme et al., 1997, 1999). When a person overestimates the benefit of or underestimates the risk of certain events it increases the likelihood of their participation in a risky activity (Byrnes, 1998; Steinberg, 2004, 2005). In short, decision theorists generally believe that by adopting a risk-benefit analysis, one might obtain the net expectancy of the risky event, which might provide useful information in predicting an individual’s involvement in risk-taking behavior (Steinberg, 2005). Under the decision-making model, risk perceptions play a fundamental role in behavioral intervention programs that try to encourage adolescents to recognize and acknowledge their own vulnerability to negative outcomes.

School bullying involves repeated aggression toward those who are perceived as weaker and less dominant and it has negative consequences for both bullies and victims (Ttofi et al., 2012). Many factors play an important role in school aggression: individual differences on how one encodes and emotionally processes evocative situations, interpretation and response to the behaviors of others, and motivation toward obtaining reward and avoiding punishment (e.g., Crick and Dodge, 1996). Another view of risk-taking behavior highlights the role of affective intensity and sensitivity to reward. According to this perspective, risky behavior cannot be fully explained by a deficiency in comprehending the potential consequences of these actions (Reyna and Farley, 2006). Moreover, leading environmental cues may “win” over cognitive control in emotionally charged circumstances. This model is supported by epidemiological reports of heightened affective responsiveness and incentive-based behavior changes when cognitive-based measures are hampered. Steinberg (2010) examined the social-neuroscience model of risk taking using behavioral measures. Steinberg used a gambling task to capture the reward-related decision-making process of participants and concluded that a heightened reward sensitivity and impulsivity were seen consistently and clearly in high-risk takers.

### Study Objectives

In sum, while many studies on bullying and its associations with a variety of risk-taking behaviors have been performed, it is surprising that no study has examined and compared the risk-taking pattern among bullying groups (i.e., bully, victim, and bully victim). This study investigated the link between risk taking and bullying in adolescents in hopes to elucidate the potential relationship between bullying and risk-taking behavior by addressing the two models of risk taking. Within the framework of these two models, this study hopes to offer a holistic model for understanding and differentiating a risk-taking pattern among the three bullying groups. Particularly, this study adopted a risk-appraisal questionnaire to address a cognitively focused process and a computerized gambling task to measure a motivational, emotion-focused process. It was hypothesized that bullies and victims would exhibit different patterns of risk appraisal and propensity. Moreover, bully victims were expected to present co-morbid patterns of both bullies and victims.

## Materials and Methods

### Participants

The study sampled students from Hong Kong public secondary schools. A multi-stage sampling procedure was used to obtain the sample. Mass invitation has been sent to all public secondary schools in Hong Kong. Schools to be studied were then randomly selected such that the proportion of students in each selected district represented the number of students in that area. This selection procedure resulted in the selection of 4 schools respectively with one from Hong Kong Island, one from Kowloon Peninsula, and two from the New Territories. As schools were given the option to withdraw from participation, a further four schools were selected by the same procedure to act as a back-up. Schools that withdrew from participation were replaced by schools on the back-up list from within the same district. Of the originally selected schools, none withdrew from participation. From each school, six students were randomly selected from grade 7 to grade 12, such that a total of 36 students were selected per school for participation. With eight students dropped out in the middle of the study, 136 adolescents were recruited and tested. Participants ranged in age from 12 to 17 years (*M* = 14.45, *SD* = 1.614) and comprised of 65 males (M) (47.8%) and 71 females (F) (52.2%). Based on self-reported experience of school bullying and victimization in the last 4 months, the participants were categorized into four distinct groups: control group (*n* = 52, *M* = 19, *F* = 33), bully group (*n* = 27, *M* = 10, *F* = 17), victim group (*n* = 20, *M* = 10, *F* = 10), and bully victim group (*n* = 37, *M* = 26, *F* = 11). All participants had a clean medical history; spoke Cantonese as their first language; had normal intelligence; and had no suspected brain damage or neurological, sensory, or psychiatric problems.

### Materials

This study consisted of a screening phase and an assessment phase. During the screening phase, participants were administered a standard Raven Progressive Matrices (RPM) as a proxy of intelligence in order to rule out low intellectual functioning. Only students with normal intelligence were invited to the assessment phase. During the assessment phase, the Computerized Cambridge Gambling Task (CGT) was administered individually to each participant. Participants then completed a demographic questionnaire including questions about age, educational level, family income, and the Cognitive Appraisal of Risky Events Questionnaire (CARE). The assessment phrase lasted approximately 1 h. All assessments were conducted by well-trained research assistants with undergraduate degrees in psychology who had been trained for approximately 2 h in test administration. All parents and adolescents provided informed consent to participate in this study. Ethical approval for the research was gained through the Human Research Ethics Committee at the Education University of Hong Kong.

### Measures

#### Non-verbal Intelligence

A traditional RPM (Raven et al., 2000) was employed in this study for screening purposes. It is a non-verbal, intellect measure that captures both analytic reasoning and visual-spatial reasoning ability. It comprises 60 items. Participants first see one implicitly meaningful diagram with one missing piece and six choices. The goal is to complete the diagram by identifying the correct piece. As the test proceeds, participants will face tougher decisions with additional implicit rulings, diagrams, and similar answers introduced. Only participants who score in the 80th percentile or above re selected and proceed to the assessment phase. Since items serves as simple, perceptual-motor control it is capable to provide a pure measure of “g factor” (deductive and reproductive ability) regardless of cultural and knowledge influence (Prabhakaran et al., 1997).

#### School-Bullying

A school-bullying questionnaire (Ng and Tsang, 2008; Wong et al., 2008) was used in this study for grouping and measuring the frequency of being bullied or victimized. The questionnaire comprised three subscales: (a) the witness, (b) the bully, and (c) the victim, which include identical items with only a difference in perspective wordings (i.e., witnessed, bullied, or victimized). Only the bully and victim subscales were administrated in this study (see **Appendix A**). Each subscale measured the rate of bullying with five categories: physical, verbal, relational, extortion, and intimidation on a 4-point frequency scale consisting of 0 (*never*), 1 (*at least once per month*), 2 (*once per week*), and 3 (*everyday*). Participants were placed into corresponding groups according to features of bullying identity. The control group comprised participants who did not indicate any bullying-related experience, the bully group comprised participants who indicated experience in bullying only, the victim group comprised participants who indicated experience in being victimized only, and the bully victim group comprised participants who indicated experience with both bullying and victimization. The bully and victim subscales had an internal reliability of α = 0.72 and 0.73, respectively.

#### Cognitive Appraisal in Risky Behavior

The CARE questionnaire was administered to measure adolescents’ outcome expectancies about the risks and benefits associated with involvement in risky activities. These outcome expectancies were measured by three CARE subscales: expected benefits (CARE_EB, **Appendix B**), expected risks (CARE_ER, **Appendix C**), and expected involvements (CARE_EI, **Appendix D**) (Fromme et al., 1997). The CARE_EB and CARE_ER scales capture the extent that participants anticipate positive or negative outcomes from their participation in 30 risky activities on a 7-point Likert scale ranging from 1 *(not likely*) to 7 (*very likely*). The CARE_EI scale uses the same Likert scale; however, it measures the likelihood of participation in these risky activities. The CARE questionnaire covers activities of illicit drug use (e.g., smoking marijuana), aggressive illegal behavior (e.g., slapping someone), risky sexual activities (e.g., sex with multiple partners), heavy drinking (e.g., drinking more than 5 alcoholic beverages), high-risk sports (e.g., mountain climbing), and negative academic work behavior (e.g., missing class). The goal of the CARE questionnaire is to predict behavioral problem tendencies associated with cognitive factors. Its reliability is excellent (Cronbach’s α for CARE_EB, CARE_ER, and CARE_EI are 0.90, 0.90, and 0.89, respectively).

#### Behavioral Measure in Risky Behavior

The CGT was administered to capture risk-taking propensity and reward-related sensitivity under uncertainty (Edwards, 1957; Andrew and Cronin, 1997; Rogers et al., 1999; Greene et al., 2000; Ladouceur et al., 2000). It aims to minimize both the learning and executive/working memory demands on participants, which can confound the interpretation of test scores. In the CGT, participants see 10 boxes colored either red or blue on the top of the screen. Display of the boxes represents the probability of winning (e.g., 9B:1R, 5B:5R). The goal is to bet on the color with a higher probability in later trials staring with 100 points to earn more points. At the beginning of the first trial, all participants were informed that they were playing for a joint school competition and those who scored in the top ten would receive a souvenir as a reward. Four trials were administered and the first and third trial was for practice/instruction for the upcoming trial. An ascending rule was implemented on the first two trials as the amount-to-bet was determined progressively by the points the participant held: 5, 25, 50, 75, and 95%. A descending rule was also implemented as participants could decrease their bet progressively. Points available to bet would grow larger or shrink smaller, respectively. A high/low pitched sound was prompted informing participants if they won or lost at the end of every round. When the participant finished a trial, his/her final points were presented allowing him/her to compare their current score with their previous score and motivate him/herself to perform better in the next trial.

##### Verbal instruction

Participants were instructed that they could see a row of boxes across the top of the screen with “X” red boxes and “Y” blue boxes. The computer had hidden a yellow token under one of these boxes. Participants had to decide whether they thought it was hidden under a red box or a blue box.

##### Ascending stage

Participants were instructed that they were provided with 100 points to start. After they chose red or blue, they had to bet a certain amount of points that they could win. The first bet they were shown was small; however, as they waited, the bets grew larger, which allowed them to choose the size of your bet. The size of the bets also depended on how many points they had: the smallest bet as always 5% of the total and the largest bet was always 95% of the total. They were encouraged to try to make as much as they could. At the final score screen in between the blocks, several prompts were used depending on how well the participants’ score was increasing. For example, “Well done! That was good. Now you are going to start off with 100 points again and you need to try to build up as many points as you can again.” If the final score got too low participants received a prompt such as “hard luck!”

##### Descending training and test

This time, the way participants’ selected their bets was slightly different as the first bet offered was large and then they gradually got smaller. Participants were instructed to practice and to make as much as they could.

##### Propensity outcome measures

The CGT consisted of six propensity outcome measures: (a) *Deliberation time* is the mean decision latency of participants choosing what color to bet on after presentation of the colored boxes. Higher scores represented longer deliberation time measured in milliseconds. (b) *Delay aversion* measures the difference in the bet’s percentage in the ascending verses the descending condition. If participants were unwilling or unable to wait to make a decision then they would be more likely to bet larger amounts when the possible bets were displayed in descending rather than ascending order. Higher scores indicated greater impulsivity. (c) *Quality of decision-making* is the proportion of trials that the participant chose to gamble on the more likely outcome. Higher scores meant that more choices were made on the likely outcome. (d) *Risk taking* is the mean proportion of points that the participant makes on each trial when they had chosen the more likely outcome. Higher score indicated that more points were placed on the likely outcome. (e) *Risk adjustment* is the degree that a participant varies their risk taking in response to the ratio of red to blue boxes on each trial. Higher score represented more likelihood to modify his/her response when the outcome probability changed. (f) *Overall proportion bet* measured the mean proportion of points bet across all trials. Higher scores represented a larger overall bet amount.

Data were analyzed with the SPSS version 21 for Windows, and were inspected for normality to ensure that the assumptions of parametric statistics were met before analyses were performed. The significance level was set at *p* < 0.05.

## Results

### Descriptive Statistics

**Table 1** summarizes the descriptive statistics for each group. Close to a marginally significant level of group differences were found in general intellectual ability [*F*(3,125) = 2.58, *p* = 0.06, η_p_^2^ = 0.06]. *Post hoc* pairwise comparisons revealed that the control group had significantly outperformed the victim group (*p* < 0.05). No significant difference was found between the control and the bully victim group (*p* = 0.68). The four groups did not differ in age, education level (self, father, and mother), and average family income (all *ps* > 0.10). Total bully scores and victim scores captured from the school bullying questionnaire are also summarized in **Table 1** with higher scores representing more frequent bullying or victimization behavior. The bully victim group showed significant higher bullying frequency than the bully group (*p <* 0.001); however, there was no difference in victim score between the victim and bully victim groups (*p >* 0.10). For sex, a chi-square analysis was applied and a statistical significance was found [see **Table 2**, χ^2^(3, *N* = 136) = 9.72, *p* = 0.02].

**Table 1:** Characteristics of controls, bullies, victims, and bully victims.

	Control (0)	Bully (1)	Victim (2)	Bully (3)				
	*n* = 52	*n* = 27	*n* = 20	*n* = 37	*F*	*P*	η_p_^2^	*Post hoc*
Age	14.56 (1.51)	14.41 (1.69)	14.35 (1.57)	14.38 (1.77)	0.13	0.94	0.00	
General intellectual ability^1,2^	108.35 (14.02)	102.60 (12.28)	100.25 (11.17)	107.18 (11.99)	2.58	0.06	0.06	0,3 > 1,2
Education level^3^	8.92 (1.37)	8.81 (1.47)	8.70 (1.81)	8.95 (1.49)	0.15	0.93	0.00	
Edu level father^3^	8.15 (2.94)	9.00 (2.39)	8.33 (3.09)	7.50 (3.29)	1.04	0.38	0.03	
Edu level mother^3^	8.53 (2.96)	8.89 (3.09)	9.47 (1.33)	8.68 (2.93)	0.47	0.71	0.02	
Average family income	6.20 (4.45)	5.92 (3.91)	5.79 (3.78)	4.65 (2.12)	1.20	0.31	0.03	
Total bully	0.00 (0.00)	1.67 (0.96)	0.00 (0.00)	2.54 (1.41)	79.39	0.00^∗∗^	0.64	
Total victim	0.00 (0.00)	0.00 (0.00)	2.85 (2.87)	2.78 (2.45)	30.80	0.00^∗∗^	0.41	

*^*1*^Standard Raven Progressive Matrices; ^*2*^Scaled score; ^*3*^American Grade System; Edu, education; ^∗^*p* < 0.05, ^∗∗^*p* < 0.01.*

**Table 2:** Cross-tabulation of sex according to bully groups.

	Control (0)	Bully (1)	Victim (2)	Bully victim (3)	Total	χ^2^	Φ
	*n* = 52	*n* = 27	*n* = 20	*n* = 37			
Male	19 (24.5)	10 (12.7)	10 (9.4)	25 (17.4)	64	9.72^∗∗^	0.27
Female	33 (27.5)	17 (14.3)	10 (10.6)	12 (19.6)	72		
Total	52	27	20	37	136		

*Number in parentheses is total number of conferences in each category. Number in cells is expected counts. ^∗^*p* < 0.05, ^∗∗^*p* < 0.01*

### Cognitive Appraisal of Risky Events

To examine whether bullying identity (control, bully, victim, bully victim) was significantly associated with the three outcome expectancies measured by CARE, a series of 2 (bully vs. not bully) × 2 (victim vs. not victim) between subject ANCOVAs with general intellectual ability as covariate were computed and summarized in **Table 3**. A *post hoc* Fisher’s Least Significant Difference (LSD) for group comparison within each sub-questionnaire was also conducted. Based on the framework of the decision-making model, a hierarchical regression analysis was conducted to examine the expected net (CARE_EN) (CARE_EN = CARE_EB – CARE_ER) in predicting CARE_EI (see **Table 4**). Results showed that the aggregate effect of expected benefit minus expected risk significantly predicted the expected involvement of risky events (*p* < 0.001).

**Table 3:** Cognitive appraisal of controls, bullies, victims, and bully victims: expected benefits, risks, and involvement.

	Control (0)	Bully (1)	Victim (2)	Bully victim (3)	
	(*n* = 51)	(*n* = 25)	(*n* = 20)	(*n* = 33)		Main effect			Interaction	
	Total	Total	Total	Total	Bully		Victim		Bully^∗^Victim		ANCOVA	
Cognitive Appraisal	*M (SD)*	*M (SD)*	*M (SD)*	*M (SD)*	*F*	*η_p_^2^*	*F*	η_p_^2^	*F*	η_p_^2^	*F*	*Post hoc*
Expected Benefits	52.65 (14.04)	59.04 (24.50)	73.20 (23.49)	82.09 (32.75)	2.98	0.02	24.49^∗∗^	0.17	0.04	0.00	11.78^∗∗^	3,2 > 1,0
Expected Risks	173.00 (36.53)	175.36 (34.29)	137.05 (46.72)	162.00 (18.78)	4.41^∗^	0.03	14.12^∗∗^	0.10	2.13	0.02	5.63^∗∗^	0,1,3 > 2
Expected Involvement	45.94 (10.11)	55.72 (17.71)	58.90 (13.44)	62.48 (18.52)	5.79^∗^	0.05	13.51^∗∗^	0.10	1.98	0.02	10.11^∗∗^	3,2,1 > 0

*^∗^*p* < 0.05, ^∗∗^*p* < 0.01*

**Table 4:** Hierarchical regressions predicting expected involvement in the cognitive appraisal of risky events.

Step	IV	Total *R*^2^	*R*^2^ change	Standardized β	*t*
1	Expected Net (EB-ER)	0.08	0.08	0.29	3.49^∗∗^

*EB, expected benefits; ER, expected risks, ^∗^*p* < 0.05, ^∗∗^*p* < 0.01*

#### Expected Benefits

The positive expectancy for risky activities was measured by the CARE_EB subscale. Higher scores represented a greater degree of positive outcome anticipation. There was significant main effect of victim [*F*(1,120) = 24.49, *p* < 0.001, η_p_^2^ = 0.17] meaning that victims anticipated risky events significantly more beneficial than the other groups did. Neither the main effect of being a bully [*F*(1,120) = 2.98, *p* = 0.09, η_p_^2^ = 0.02] nor the interaction between being a bully and victim [*F*(1,120) = 0.04, *p* = 0.83, η_p_^2^ = 0.00] was significant. Inspection of means showed bully victims and victims scored significantly higher than controls and bullies, who confirmed a main effect of victims overestimating the benefits of risky events (see **Table 3**).

#### Expected Risks

The negative expectancy of risk activities was captured through the CARE_ER subscale. Higher scores represented a greater degree of anticipating negative outcomes. There were significant main effects of bully [*F*(1,120) = 4.41, *p* < 0.05, η_p_^2^ = 0.03] and victim [*F*(1,120) = 14.12, *p* < 0.001, η_p_^2^ = 0.10] meaning that participants with a bully or a victim identity anticipated risky events as significantly more or less risky. There was no significant interaction effect (*p* = 0.15). Mean scores of victims were significantly lower than the other groups suggesting a main effect of victims underestimating risks and bullies overestimated risks (**Table 3**).

#### Expected Involvements

Anticipation of participation for risky events was measured using CARE Expected Involvement subscale. The higher score suggests the higher probability of participation in potentially harmful activities. There were significant main effects of Victim [*F*(1,120) = 14.27, *p* < 0.001, η_p_^2^ = 0.10] and Bully [*F*(1,120) = 5.79, *p* < 0.05, η_p_^2^ = 0.05]. There was no significant interaction effect (*p* = 0.16). Mean scores on Bully, Victim, and Bully Victim were significantly higher than Control group (see **Table 3**). The pattern suggested participants with Bully or Victim identity anticipate higher expected involvement in risky events.

### Predicting Bully and Victim Scores

A hierarchical regression analysis was conducted to examine what CARE variables best predicted the severity of bullying and victimization. **Table 5** shows that CARE accounted for an additional 16.9% of the variance in Bullying severity, and CARE_EI was the only significant predictor of Bullying severity (β = 0.40, *t* = 4.30, *p* < 0.001). Regarding the severity of victimization, **Table 6** shows that CARE accounted for only an additional 2.5% of the variance in victimization severity, and none of the CARE variables significantly predicted the frequency of being bullied.

**Table 5:** Hierarchical regressions predicting cognitive appraisal in risky events variables among bully total.

Step	IV	Total *R*^2^	*R*^2^ change	Standardized β	*t*
1	Expected Involvements	0.169	0.169	0.365	4.296^∗∗^
	Expected Benefits			0.111	1.25
	Expected Risks			0.042	0.499

*^∗^*p* < 0.05, ^∗∗^*p* < 0.01*

**Table 6:** Hierarchical regressions predicting cognitive appraisal in risky events variables among victim total.

Step	IV	Total *R*^2^	*R*^2^ change	Standardized β	*t*
1	Expected Benefits	0.025	0.025	0.111	1.157
	Expected Involvements			0.086	0.930
	Expected Risks			0.033	0.366

*^∗^*p* < 0.05, ^∗∗^*p* < 0.01*

### Behavioral Measure in Risky Behavior: The Cambridge Gambling Task

In order to capture behavior differences in adolescents’ reward-related sensitivity and risk-taking propensity, the six CGT factors previously listed were analyzed with general intellectual ability as a covariate using a 2 (bully vs. not bully) × 2 (victim vs. not victim) between-subjects ANCOVA (see **Table 7**). A one-way ANCOVA followed by a *post hoc* LSD were conducted to examine significant differences between groups within each factor. Correlations between the CGT factors are shown in **Table 8**.

**Table 7:** Cognitive appraisal of controls, bullies, victims, and bully victims using the Cambridge Gambling Task (CGT).

	Control (0)	Bully (1)	Victim (2)	Bully victim (3)
	(*n* = 51)	(*n* = 25)	(*n* = 20)	(*n* = 33)		Main effect			Interaction	
	Total	Total	Total	Total	Bully		Victim		Bully^∗^Victim		ANCOVA
Cambridge Gambling Task	*M (SD)*	*M (SD)*	*M (SD)*	*M (SD)*	*F*	η_p_^2^	*F*	η_p_^2^	*F*	η_p_^2^	*F*	*Post hoc*
Deliberation time	2404.49 (970.58)	2618.58 (111.80)	1865.76 (409.84)	2317.85 (1274.03)	3.27	0.03	5.56^∗^	0.04	1.25	0.01	2.60	0,1,3 > 2
Delay aversion	0.26 (0.32)	0.21 (0.29)	0.46 (0.25)	0.30 (0.22)	3.30	0.03	7.41^∗∗^	0.06	0.85	0.01	3.04^∗^	2 > 0,1,3
Quality of decision making	0.88 (0.14)	0.88 (0.10)	0.93 (0.11)	0.86 (0.16)	1.75	0.01	0.68	0.01	5.64^∗^	0.04	2.30	2 > 0,1,3
Risk taking	0.53 (0.14)	0.49 (0.13)	0.52 (0.14)	0.54 (0.17)	0.13	0.00	0.92	0.01	1.20	0.01	0.73	
Risk adjustment	0.81 (0.97)	1.15 (0.91)	0.40 (0.91)	1.11 (0.79)	9.66^∗∗^	0.07	1.50	0.01	0.45	0.00	3.28^∗^	1,3 > 0,2
Overall bet proportion	0.49 (0.14)	0.45 (0.13)	0.49 (0.13)	0.50 (0.15)	0.48	0.00	1.39	0.01	0.42	0.00	0.68	

*^∗^*p* < 0.05, ^∗∗^*p* < 0.01*

**Table 8:** Correlations among CGT variables.

	Delay aversion	Quality of decision making	Risk taking	Risk adjustment	Overall bet proportion
Deliberation time	-0.14	-0.49^∗∗^	-0.24^∗∗^	-0.05	-0.23^∗∗^
Delay aversion		0.03	0.08	-0.08	0.13
Quality of decision making			0.27^∗∗^	0.19^∗^	0.32^∗∗^
Risk taking				-0.09	0.97^∗∗^
Risk adjustment					-0.15

*^∗^*p* < 0.05, ^∗∗^*p* < 0.01*

#### Deliberation Time

For mean decision latency, there was a significant main effect of victim [*F*(1,120) = 5.56, *p* < 0.05, η_p_^2^ = 0.04], and a marginal main effect of bully [*F*(1,120) = 3.27, *p* = 0.07, η_p_^2^ = 0.03]. An interaction effect was not found [*F*(1,120) = 1.25, *p* = 0.27, η_p_^2^ = 0.01]. Inspection of means revealed that victims might use shortened decision times than bullies (see **Table 7**).

#### Delay Aversion

Delay aversion captured differences between the scores in the descending and ascending conditions. There was a marginal main effect for bully [*F*(1,120) = 3.30, *p* = 0.07, η_p_^2^ = 0.03] and a significant main effect for victim [*F*(1,120) = 7.41, *p* < 0.01, η_p_^2^ = 0.06]. An interaction effect was not found [*F*(1,120) = 0.85, *p* = 0.36, η_p_^2^ = 0.01]. Inspection of mean scores on delay aversion revealed that victims had higher scores than controls, bullies, and bully victims, suggesting that victims might be more impulsive than the other groups.

#### Decision-Making Quality

Neither a main effect of bully [*F*(1,120) = 1.75, *p* = 0.19, η_p_^2^ = 0.01] nor a main effect of victim [*F*(1,120) = 0.68, *p* = 0.41, η_p_^2^ = 0.01] were significant. There was a significant interaction effect of bully and victim [*F*(1,120) = 5.64, *p* < 0.05, η_p_^2^ = 0.04]. The interaction effect revealed that with the absence of bullies, victims alone incurred significant impact to the proportion of trials participants gambled on the more favorite outcome, leaving the victim group as the only group that made more choices on the likely outcome. A subsequent means scores comparison revealed that victims scored higher than bully victims and controls (**Table 7**).

#### Risk Taking

Neither a main effect of bully (*p* = 0.46) or victim (*p* = 0.85) nor any interactions were significant (*p* > 0.10).

#### Risk Adjustment

There was a significant main effect of Bully [*F*(1,120) = 9.66, *p* < 0.01, η_p_^2^ = 0.07]. Neither main effects of Victim [*F*(1,120) = 1.50, *p* = 0.22, η_p_^2^ = 0.01] nor interaction effect [*F*(1,120) = 0.45, *p* = 0.50, η_p_^2^ = 0.00] were significant. Inspection of mean scores revealed that Bully had higher risk adjustment scores than Victim (**Table 7**) suggesting Bully identity exhibited higher risk modulation.

#### Overall Bet Proportion

There was no significant main effect of bully [*F*(1,120) = 0.48, *p* = 0.49, η_p_^2^ = 0.00], victim [*F*(1,120) = 1.39, *p* = 0.24, η_p_^2^ = 0.001], or interaction [*F*(1,120) = 0.42, *p* = 0.52, η_p_^2^ = 0.00].

### Predicting Bullying and Victim Scores

A hierarchical regression analysis was conducted to examine what CGT variables best predicted the severity of bullying and victimization. **Table 9** shows that CGT accounted for an additional 10.2% of the variance in bullying severity and risk adjustment was the only significant predictor of bullying severity (β = 0.25, *t* = 2.77, *p* < 0.01). Regarding the severity of victimization, **Table 10** shows that CGT only accounted for an additional 3.5% of the variance in the frequency of victimization and none of the CGT variables significantly predicted the severity of victimization.

**Table 9:** Hierarchical regressions predicting CGT variables among bully total.

Step	IV	*R*^2^	*R*^2^ change	Standardized β	*t*
1	Risk adjustment	0.102	0.102	0.252	2.770^∗∗^
	Quality of decision making			-0.131	1.239
	Risk taking			0.343	0.923
	Delay aversion			-0.041	0.467
	Deliberation time			-0.043	0.431
	Overall proportion			-0.124	0.324

*^∗^*p* < 0.05, ^∗∗^*p* < 0.01*

**Table 10:** Hierarchical regressions predicting CGT variables among victim total.

Step	IV	Total *R*^2^	*R*^2^ change	Standardized β	*t*
1	Quality of decision making	0.035	0.035	-0.086	0.782
	Risk adjustment			0.073	0.776
	Delay aversion			0.070	0.775
	Deliberation time			-0.050	0.488
	Risk taking			0.085	0.222
	Overall proportion			0.078	0.196

*^∗^*p* < 0.05, ^∗∗^*p* < 0.01*

## Discussion

### Risk Taking in Bullies

Bullying and risk-taking behavior are two common problems among adolescents worldwide and can cause serious impact to adolescents’ physical, psychological, social, and educational functioning when both coexist in the same youth (Currie et al., 2012). Despite the associations between bullying and risk-taking behaviors, no study thus far has examined and compared the risk-taking pattern among bullying groups (i.e., bully, victim and bully victim). This study elucidated the risk-taking pattern in these three bullying groups by addressing two risk-taking models (i.e., the decision-making model and the social-neuroscience model) and clarifying how adolescents’ characteristics in risk taking may associate with outcomes in bullying or victimization.

These findings suggest that being a bully or victim is associated with distinct results on the two approaches, and these results contribute several novel findings to the current literature. Under the decision-making framework, bullying was associated with higher negative expectancy on risky activities and more expected involvement in them. In other words, being a bully meant more anticipated negative outcomes in risky activities. Interestingly, despite the higher anticipation of negative outcomes, bullies expected themselves to have higher involvement in these activities compared to the non-bully groups. In CGT, bullying was associated with positive risk-taking adjustment, an increased amount of gambling their points when the odds were in their favor, and vice versa. These results indicated that being a bully was vigilant in situational deliberation and risk modulation, and contradicted the general agreement that there was a positive link between bullying and impulsivity (Olweus, 1995; Schwartz et al., 2001; O’Brennan et al., 2009). Perhaps bullying is a unique, complex form of interpersonal aggression or group phenomenon (e.g., Olweus, 2001; Salmivalli, 2001; Rodkin and Hodges, 2003) and bullies tend to be hyper-vigilant to social cues and attribute negative intentions to others (Vaillancourt et al., 2003). The paradox of aggression is that it is both adaptive and maladaptive. Aggressive adolescents are at risk for a host of possible negative consequences (e.g., disciplinary punishment, school suspension, etc.) (Dodge et al., 2006). However, aggression is often a successful means in changing other’s behavior and can be used to acquire resources and maintain group boundaries. Moreover, researchers generally agree that bullies tend to exhibit high levels of social intelligence and the ability to manipulate peers (e.g., Peeters et al., 2010). In other words, bullies may be a group of adolescents who are sensitive and respond quickly to external cues through peer manipulation, resulting in potential risk for themselves and victims.

### Risk Taking in Victims

The results of the CARE questionnaire suggested that victims were associated with an overestimation of benefits, an underestimation of risks, and higher expected involvement in risky events. According to the decision-making model, these patterns suggested the lowest sense of risk and the highest vulnerability to it (Hunter and Boyle, 2004; Hunter et al., 2004; Wachs et al., 2012). Motivationally, victims were associated with less deliberation time and more delay aversion in CGT. In CGT, even though delay response does not increase the available information for decision making, shorter deliberation time may indicate impulsive decision-making. This assumption was further supported by their poor performance in delay aversion and that victims were more likely to bet larger amounts when bets were displayed in a descending rather than an ascending way. It is well established that impulsivity is particularly relevant to peer victimization. For example, children with attention-deficit/hyperactivity disorder who share common features of impulsivity and high risk-taking propensity, show marked impairment in peer relationships and are significantly bullied by peers (e.g., Hoza, 2007). Researchers argued that individuals who are low on self-control or high on impulsivity are unable to see the consequences of their actions and more likely to place themselves in risky situations without regard for their long-term outcome (e.g., Higgins et al., 2009).

### Risk Taking in Bully Victims

Consistent with our hypothesis, bully victims presented the combined pattern of the two pure groups. Cognitively, bully victims showed similar, but not identical patterns of the two pure groups. Bully victims overestimated the benefits of risk as well as benefit and projected the highest possibility of engaging in risky behavior. Motivationally, this unique group shared identical patterns with bullies in terms of positive risk adjustment. In other words, bully victims exhibited similar risk tendencies to victims in terms of cognitive-focused processes and to bullies on emotion-focused processes. Importantly, compared with bullies, bully victims had significantly higher bullying scores, showing that they engaged in a wider range of more frequent bullying activities. In fact, previous studies that focused on the frequency and forms of bullying also reported that bully victims used wider and more aggressive strategies than the pure groups did (Olweus, 1993; Schwartz, 1999; Olafsen and Viemerö, 2000). Additional evidence has suggested that bully victims may exhibit the highest aggression level of all three groups as well as the poorest emotion regulation (e.g., Nansel et al., 2004). Olweus (1978) suggested that bullies were generally more functional, more likely to use proactive aggression, and more likely to have an extensive social network than bully victims, who were more likely to react aggressively and show troubling risk patterns across virtually all adjustment indicators.

### Implications

Concern about the frequency and effects of adolescent bullying was reflected in the increase in research aimed at understanding its causes and consequences in order to develop appropriate policy and intervention strategies. Nonetheless, the success of intervention programs to prevent or mitigate bullying in adolescence has been limited (e.g., Olweus, 1999; Merrell et al., 2008). Even when programs have an impact, the improvement appears to be in changing adolescents’ knowledge and perceptions on bullying, but not the behavior. This study extended the existing literature on bullying and victimization by addressing the co-occurrence of bullying and victimization and identifying a range of risk-taking patterns associated with the three groups. This study suggested that there were significant group differences on how bullies, victims, and bully victims appraised risky events and emotionally processed evocative risky situations. Moreover, this study identified different predictors of bullying and victimization. In accordance with the decision-making model, this study provided further support that participants’ perceived benefits and risk may play a key role in predicting their expected involvement in risky behaviors. Given that expected involvement in risky events is a significant predictor to the severity of bullying, future intervention programs should include cognitive training in risk evaluation (Steinberg, 2007). For instance, presenting strategies for effective risk and benefit evaluation will encourage less risky and healthier choices. Moreover, given the high vigilance of bullies to external cues, future interventions should target the enhancement of social skills and coping strategies on peer conflicts. Victims with marked impulsivity, on the other hand, may be targeted to deliver interventions on self-control that strengthen their ability to fit in with their peers and reduce the likelihood of rejection and victimization, or to provide supportive interpersonal relationships to reduce their isolation (Andreou et al., 2005). Effective and comprehensive evaluation of risk is another intervention focus for victims to help increase their awareness on the consequences of their risky choices.

### Limitations and Future Directions

This study has several limitations that should be considered when interpreting our findings. First, the sample size was relatively small. Future studies should include a larger sample size and a power calculation to determine what sample size would be needed to detect differences between groups. Second, this study was cross-sectional in nature. Therefore, causation cannot be determined. A temporal relationship between bullying and a risk-taking pattern could not be inferred. Questions of causation can only be answered in a longitudinal research design, which should be implemented in the future. Third, this study was localized to Chinese adolescents. Even though assessment tools were standardized, a comprehensive assessment tool on risk appraisal may be needed to elicit cultural differences on risk perception. In China, school bullying is often regarded as a collective act (Cheng et al., 2011; Huang et al., 2013; Chui and Chan, 2015), and whether this is a form of “collective bullying” that may cause an impact to the risk-taking pattern is yet to be determined. Fourth, although this study examined emotional aroused stimulation (i.e., the gambling task), it might not present an authentic context where bullying is most likely to occur. Lastly, this study was limited by its use of self-report measures (e.g., the bully or the victim). Biases such as social desirability and retrospective recall issues may occur. Moreover, the likelihood of under-reporting adolescents’ school bullying behavior was also possible. Future research should include other bullying behavioral assessments such as peer and teacher nominations and behavioral observations as supplementary measures to validate self-reported findings (e.g., Espelage and Holt, 2001; Espelage and Swearer, 2003).

With growing recognition that bullying is a complex phenomenon that is influenced by multiple factors, past research has been examined within a sociological framework. This study enhanced the understanding of this social phenomenon from a psychological perspective by examining the risk-taking pattern associated with bullies, victims, and bully victims. This analysis will aid in not only comprehending the mechanism of how these groups respond to risk taking, but also lead to improved practice for prevention and intervention. Overall, bullying behavior is the result of the interaction of multiple causes and factors, and it is too early to believe that the identification of various risk-taking patterns or specific intervention that target their unique risk-taking processes would be a cure-all solution for the elimination of bullying or victimization in these groups. However, this study provides a basis for future studies to adopt a holistic approach in investigating the link between bullying and risk-taking behavior.

## Author Contributions

KP is the sole author of this research article and her task involves conception or design of the work, data analysis and interpretation, drafting the article and final approval of the version to be published.

## Conflict of Interest Statement

The author declares that the research was conducted in the absence of any commercial or financial relationships that could be construed as a potential conflict of interest.

## APPENDIX A

### School Bullying Questionnaire

**Table:**

		**Never**	**At least once a month**	**About once a week**	**Everyday**
1	During the past 4 months, have you physically bullied others, such as kicking, punching, slapping, and pushing around?	0	1	2	3
2	During the past 4 months, have you verbal bullied others, such as cursing, ridiculing, insulting, and calling hurtful nicknames?	0	1	2	3
3	During the past 4 months, have you relational bullied others, such as ignoring, spreading rumors, and excluding from social circle?	0	1	2	3
4	During the past 4 months, have you extorted others, such as extorting, blackmailing, and exacting?	0	1	2	3
5	During the past 4 months, have you intimidated and forced others, such as intimidating, threatening, and scaring victims?	0	1	2	3
6	During the past 4 months, have you been physically bullied by your peers, such as being kicked, punched, slapped, and pushed around?	0	1	2	3
7	During the past 4 months, have you been verbal bullied by your peers, such as being cursed at, ridiculed, insulted, and called hurtful nicknames?	0	1	2	3
8	During the past 4 months, have you been isolated by your peers, such as being ignored, spread rumors, and excluded from social circle?	0	1	2	3
9	During the past 4 months, have you been extorted by your peers, such as being extorted, blackmailed, and exacted?	0	1	2	3
10	During the past 4 months, have you been intimidated by your peers, such as intimidated, threatened, and scared victims?	0	1	2	3

## APPENDIX B

### Cognitive Appraisal of Risky Event Questionnaire (CARE) – Expected Benefits

On a scale of 1 (not at all likely) to 7 (extremely likely), HOW LIKELY IS IT THAT YOU WOULD EXPERIENCE SOME POSITIVE CONSEQUENCE (e.g., pleasure, win money, feel good about yourself, etc.) if you were to engage in these activities?

**Table:**

		**Positive Consequences**						
		**Not at all likely**		**Moderately likely**			**Extremely likely**	
1	Trying/using drugs other than alcohol or marijuana	1	2	3	4	5	6	7
2	Missing class or work	1	2	3	4	5	6	7
3	Grabbing, pushing, or shoving someone	1	2	3	4	5	6	7
4	Leaving a social event with someone I have just met	1	2	3	4	5	6	7
5	Driving after drinking alcohol	1	2	3	4	5	6	7
6	Making a scene in public	1	2	3	4	5	6	7
7	Drinking more than 5 alcoholic beverages	1	2	3	4	5	6	7
8	Not studying for exam or quiz	1	2	3	4	5	6	7
9	Drinking alcohol too quickly	1	2	3	4	5	6	7
10	Disturbing the peace	1	2	3	4	5	6	7
11	Damaging/destroying public property	1	2	3	4	5	6	7
12	Sex without protection against pregnancy	1	2	3	4	5	6	7
13	Leaving tasks or assignments for the last minute	1	2	3	4	5	6	7
14	Hitting someone with a weapon or object	1	2	3	4	5	6	7
15	Rock or mountain climbing	1	2	3	4	5	6	7
16	Sex without protection against sexually transmitted diseases	1	2	3	4	5	6	7
17	Playing non-contact team sports	1	2	3	4	5	6	7
18	Failing to do assignments	1	2	3	4	5	6	7
19	Slapping someone	1	2	3	4	5	6	7
20	Not studying or working hard enough	1	2	3	4	5	6	7
21	Punching or hitting someone with fist	1	2	3	4	5	6	7
22	Smoking marijuana	1	2	3	4	5	6	7
23	Sex with a variety of partners	1	2	3	4	5	6	7
24	Snow or water skiing	1	2	3	4	5	6	7
25	Mixing drugs and alcohol	1	2	3	4	5	6	7
26	Getting into a fight or argument	1	2	3	4	5	6	7
27	Involvement in sexual activities without my consent	1	2	3	4	5	6	7
28	Playing drinking games	1	2	3	4	5	6	7
29	Sex with someone I have just met or don’t know well	1	2	3	4	5	6	7
30	Playing individual sports	1	2	3	4	5	6	7

## APPENDIX C

### Cognitive Appraisal of Risky Event Questionnaire (CARE) – Expected Risks

On a scale of 1 (not at all likely) to 7 (extremely likely), HOW LIKELY IS IT THAT YOU WOULD EXPERIENCE SOME NEGATIVE CONSEQUENCE (e.g., become sick, be injured, embarrassed, lose money, suffer legal consequences, fail a class, or feel bad about yourself) if you engaged in these activities?

**Table:**

		**Positive Consequences**						
		**Not at all likely**		**Moderately likely**			**Extremely likely**	
1	Trying/using drugs other than alcohol or marijuana	1	2	3	4	5	6	7
2	Missing class or work	1	2	3	4	5	6	7
3	Grabbing, pushing, or shoving someone	1	2	3	4	5	6	7
4	Leaving a social event with someone I have just met	1	2	3	4	5	6	7
5	Driving after drinking alcohol	1	2	3	4	5	6	7
6	Making a scene in public	1	2	3	4	5	6	7
7	Drinking more than 5 alcoholic beverages	1	2	3	4	5	6	7
8	Not studying for exam or quiz	1	2	3	4	5	6	7
9	Drinking alcohol too quickly	1	2	3	4	5	6	7
10	Disturbing the peace	1	2	3	4	5	6	7
11	Damaging/destroying public property	1	2	3	4	5	6	7
12	Sex without protection against pregnancy	1	2	3	4	5	6	7
13	Leaving tasks or assignments for the last minute	1	2	3	4	5	6	7
14	Hitting someone with a weapon or object	1	2	3	4	5	6	7
15	Rock or mountain climbing	1	2	3	4	5	6	7
16	Sex without protection against sexually transmitted diseases	1	2	3	4	5	6	7
17	Playing non-contact team sports	1	2	3	4	5	6	7
18	Failing to do assignments	1	2	3	4	5	6	7
19	Slapping someone	1	2	3	4	5	6	7
20	Not studying or working hard enough	1	2	3	4	5	6	7
21	Punching or hitting someone with fist	1	2	3	4	5	6	7
22	Smoking marijuana	1	2	3	4	5	6	7
23	Sex with a variety of partners	1	2	3	4	5	6	7
24	Snow or water skiing	1	2	3	4	5	6	7
25	Mixing drugs and alcohol	1	2	3	4	5	6	7
26	Getting into a fight or argument	1	2	3	4	5	6	7
27	Involvement in sexual activities without my consent	1	2	3	4	5	6	7
28	Playing drinking games	1	2	3	4	5	6	7
29	Sex with someone I have just met or don’t know well	1	2	3	4	5	6	7
30	Playing individual sports	1	2	3	4	5	6	7

## APPENDIX D

### Cognitive Appraisal of Risky Event Questionnaire (CARE) – Expected Involvements

On a scale of 1 (not at all likely) to 7 (extremely likely), HOW LIKELY IS IT THAT YOU WILL ENGAGE IN EACH OF THESE ACTIVITIES in the next 6 months?

**Table:**

		**Positive Consequences**					
		**Not at all likely**		**Moderately likely**			**Extremely likely**	
1	Trying/using drugs other than alcohol or marijuana	1	2	3	4	5	6	7
2	Missing class or work	1	2	3	4	5	6	7
3	Grabbing, pushing, or shoving someone	1	2	3	4	5	6	7
4	Leaving a social event with someone I have just met	1	2	3	4	5	6	7
5	Driving after drinking alcohol	1	2	3	4	5	6	7
6	Making a scene in public	1	2	3	4	5	6	7
7	Drinking more than 5 alcoholic beverages	1	2	3	4	5	6	7
8	Not studying for exam or quiz	1	2	3	4	5	6	7
9	Drinking alcohol too quickly	1	2	3	4	5	6	7
10	Disturbing the peace	1	2	3	4	5	6	7
11	Damaging/destroying public property	1	2	3	4	5	6	7
12	Sex without protection against pregnancy	1	2	3	4	5	6	7
13	Leaving tasks or assignments for the last minute	1	2	3	4	5	6	7
14	Hitting someone with a weapon or object	1	2	3	4	5	6	7
15	Rock or mountain climbing	1	2	3	4	5	6	7
16	Sex without protection against sexually transmitted diseases	1	2	3	4	5	6	7
17	Playing non-contact team sports	1	2	3	4	5	6	7
18	Failing to do assignments	1	2	3	4	5	6	7
19	Slapping someone	1	2	3	4	5	6	7
20	Not studying or working hard enough	1	2	3	4	5	6	7
21	Punching or hitting someone with fist	1	2	3	4	5	6	7
22	Smoking marijuana	1	2	3	4	5	6	7
23	Sex with a variety of partners	1	2	3	4	5	6	7
24	Snow or water skiing	1	2	3	4	5	6	7
25	Mixing drugs and alcohol	1	2	3	4	5	6	7
26	Getting into a fight or argument	1	2	3	4	5	6	7
27	Involvement in sexual activities without my consent	1	2	3	4	5	6	7
28	Playing drinking games	1	2	3	4	5	6	7
29	Sex with someone I have just met or don’t know well	1	2	3	4	5	6	7
30	Playing individual sports	1	2	3	4	5	6	7
